# Epistemic Corruption, the Pharmaceutical Industry, and the Body of Medical Science

**DOI:** 10.3389/frma.2021.614013

**Published:** 2021-03-08

**Authors:** Sergio Sismondo

**Affiliations:** Department of Philosophy, Queen’s University, Kingston, ON, Canada

**Keywords:** bias, medical research, pharmaceutical industry, epistemic corruption, conflict of interest

## Abstract

When a knowledge system importantly loses integrity, ceasing to provide the kinds of trusted knowledge expected of it, we can label this *epistemic corruption*. Epistemic corruption often occurs because the system has been co-opted for interests at odds with some of the central goals thought to lie behind it. There is now abundant evidence that the involvement of pharmaceutical companies corrupts medical science. Within the medical community, this is generally assumed to be the result of conflicts of interest. However, some important ways that the industry corrupts are not captured well by standard analyses in terms of conflicts of interest. It is not just that there is a body of medical science perverted by industry largesse. Instead, much of the corruption of medical science via the pharmaceutical industry happens through grafting activities: Pharmaceutical companies do their own research and smoothly integrate it with medical science, taking advantage of the legitimacy of the latter.

## Introduction: Epistemic Corruption

“Corrupt” and its cognates are old terms with many metaphorical uses. Bodies, fruits and meats are corrupted when they begin to rot, decompose, or otherwise spoil. What is thought pure is corrupted when mixed with something foul or lesser, as when air is made foul by pestilence or smoke, noble lineages are supposedly lessened by poor marriages, or people become less good simply because of the pressures of society. “Each of us is born with a share of purity, predestined to be corrupted by our commerce with mankind, by that sin against solitude” (Cioran 2012 [1949]).

It is only a small step from the introduction of pollution to the perversion of ends, as when a public official is corrupted by money or power for a purpose, to serve some interests rather than others. This is the most familiar kind of corruption today—so common that the metaphor has largely died—in which corrupted office holders and institutions have been captured by outside interests, or perhaps serve only their own interests. Thus there is a United Nations Convention against Corruption, which never needs to explicitly define “corruption,” though it identifies it as involving a constellation of crimes that include bribery, embezzlement, influence peddling, illicit enrichment, etc., (United Nations 2004).

There can be value in analyzing knowledge systems in terms of all the above and other senses of the metaphor. When a knowledge system importantly loses integrity, ceasing to provide the kinds of trusted knowledge expected of it, or even in some cases when it ceases to establish trust, we can label this *epistemic corruption*. For example, the weaknesses of mathematical models can become entrenched, especially if they are constantly adjusted through curve-fitting, as has been claimed about several epidemiological models of the spread of Covid-19 (e.g., Jewell et al., 2020). Or, environmental toxicology may systematically lack information about the risks of a large number of industrial and agricultural chemicals, because powerful entities can control private science (e.g., on fluorinated compounds see Richter et al., 2018) and shape public science (e.g., on glyphosate, see Thacker 2019). And, outside the sciences, although many accusations of “fake news” are wide of the mark, large swaths of both social and traditional media are genuinely untrustworthy, whether because of interests that shape the creation or the dissemination of news, or because of inherent weaknesses of systems designed to capture audiences’ attention.

My focus here is on how the pharmaceutical industry corrupts medical science. Using its very substantial resources, pharmaceutical companies co-opt medical knowledge systems for their particular interests, interests that conflict with the integrity and at least some of the central goals thought to lie behind medicine. It would seem that the body of medical science is corrupted because some assumed purity—though purity is always notional—has been affected by contact with outside interests.

## Pharmaceutical Industry Affects Medical Research

For the past 25 years, researchers have been studying the effects of industry funding—most often from pharmaceutical companies—on medical science. One typical protocol compares outcomes in industry-funded and other clinical trials in some therapeutic area, or for some class of drugs or medical devices, working either from searches of the published literature or from some other sample, such as conference abstracts. Most reports of clinical trials declare sources of funding, so analysts can often cleanly divide publications and make comparisons. In addition, clinical trials within areas often have enough uniformity that at meta-analyses can sometimes be done. Since the mid-1990s, there have been hundreds of published studies of industry influence, comparing many thousands of clinical trials across all domains of medicine. The researchers designing and following these protocols often frame them as analogous to medical studies, with industry funding being the intervention, and the integrity and stability of the body of medical research being the outcome.

A 2017 Cochrane Review (Lundh et al., 2017, updated from; Lundh et al., 2012) provides a meta-analysis of such studies of industry funding, in which 75 studies, comparing more than 8,000 trials, met inclusion criteria. In all of its dimensions, the 2017 meta-analysis arrives at the same or similar results as had earlier quantitative and qualitative reviews (Bekelman et al., 2003; Lexchin et al., 2003; Schott et al., 2010). In the meta-analysis, industry funding had a risk ratio of 1.27 (95% CI: 1.17–1.51) of producing favorable efficacy results, and of 1.34 (95% CI: 1.19–1.51) of drawing favorable overall conclusions (in this study the harm results were not statistically different between industry and non-industry funding). Since there is no reason to think that non-industry funding skews results in any consistent direction, one can only conclude that industry funding biases the outcomes of clinical trials. Put simply, if a pharmaceutical company funds a trial, the chances of results and conclusions in that company’s favor are increased. However, in this study, industry and non-industry research did not differ on such standard methodological quality concerns as sequence generation, allocation concealment, follow-up, or selective outcome reporting; and industry sponsored studies even had better blinding procedures.

The authors of the Cochrane Review conclude: “Our analyses suggest the existence of an industry bias that cannot be explained by standard ‘Risk of bias’ assessments” (Lundh et al., 2017). When pharmaceutical and other companies sponsor research there is a bias—a systematic tendency toward results serving their interests—but the bias is not seen in the formal factors routinely associated with low-quality science. The implication is that industry funding itself should be considered a standard “risk of bias” factor in clinical trials, one that is quantifiable, and even quantified, and pushes in predictable directions. Industry funding affects the results of clinical trials.

## But Funding is Rarely just Funding

The Cochrane Review I have just described shows that the pharmaceutical industry corruption of medical science doesn’t happen through the mechanisms currently assessed by typical formal methodological measures. Funding itself corrupts medical science. But this does not mean that it is mysterious.

The most common way of understanding corruption through funding is in terms of conflict of interest. Perhaps funding and payments to researchers create conflicts of interest, which—for conscious or unconscious reasons—affect their actions, their judgments, and their conclusions. As a result, these conflicted researchers become more likely to report outcomes friendly to their funders. However, something else is at play here as well, and it is this that I want to illustrate below.

There is abundant evidence that conflicts of interest are important in many domains, including across medicine. For example, financial conflicts on committees producing clinical practice guidelines tend to produce assessments of evidence and recommendations that favor the companies and industries involved (Cosgrove et al., 2013; Lexchin 2020). In terms of medical practice, a recent systematic review shows that payments to physicians influence prescribing (Mitchell et al., 2020). The broad issue of conflict of interest is important enough that the United States Institute of Medicine issued a detailed report on it, overwhelmingly about how financial conflicts involving industry affect researchers’ and physicians’ judgment (Institute of Medicine, 2009). Despite such evidence, a focus on conflict of interest hides how pharmaceutical companies influence published results and outcomes.

Funding is rarely just funding. Most pharmaceutical company-sponsored clinical trials are designed, organized, audited, analyzed, and written up by the companies and their hired subcontractors. This is all work that happens behind the scenes, obscured by the form of academic publication. Thus much of the corruption can happen through more substantive medical choices and through structures of influence and control, as I describe below.

Roughly 70–75% of the industry’s expenditures on clinical trials go to contract research organizations (CROs), rather than to independent researchers in the form of grants (Mirowski and Van Horn 2005; Fisher 2008; Westrock 2016). CROs together have revenue estimated to be approximately US$50 billion in 2020, most of it coming from pharmaceutical industry clinical trials (Fortune Business Insights, 2019). As a result, in the comparison of “industry-sponsored” and independent research, in most cases the “sponsorship” involves direct control over the research.

Even when it appears that industry-sponsored trials are led by academic or other actors, and that their subjects are recruited via independent clinics, hospitals and academic medical centers, it is most likely that at a higher level they are run by CROs working for pharmaceutical companies, and analyzed by company statisticians and others. Manuscripts are most likely drafted by ghostwriters on structures created by publication planners, and then shepherded through to publication by those planners, with limited opportunities for their academic and other independent authors to contribute (Fugh-Berman and Dodgson, 2008; Sismondo 2009; Matheson 2016). The published articles, then, are largely creations of the companies, even if the nominal authors include independent researchers. All of this constitutes the “ghost-management” of medical research (Sismondo 2018).

The ghost-management of trials affords many opportunities to intervene on individual publications and to affect the published record, producing the effects of industry sponsorship I described above. I list some significant categories, for each of which I provide an example or evidence.

(a) Companies can design studies that are likely to produce favorable results, making careful choices of comparators, doses, experimental populations, surrogate endpoints, trial durations, and definitions. For example, in Merck’s testing of its COX-2 inhibitor rofecoxib, it used most of these techniques to improve one or another of its published trials (Whitstock 2018).
(b) Given the ghost-management of industry-funded research, funding almost certainly affects the interpretation of data and the writing of articles. Internal company documents and presentations show that the companies are fully aware of the opportunities for spin (e.g., Moffatt and Elliott 2007; McHenry 2010).
(c) Sometimes the corruption goes so far as to count as scientific misconduct, such as direct manipulation of data, omission of adverse events, etc. On the basis of documents from litigation against Forest Laboratories for misleading marketing of citalopram, Jureidini et al. (2016) establish conclusively that the ghost-management of the research allowed company employees to publish efficacy and safety conclusions that were inconsistent with what the trial data could support.
(d) Industry trials with positive results are over-represented in the medical journals, and those with negative results are under-represented, resulting in significant publication biases. In antidepressant trials submitted to regulatory agencies such as the United States Food and Drug Administration (Turner et al., 2008) or the Swedish regulatory agency (Melander et al., 2003)—and thus all industry trials—positive results are much more likely to be published. The positive trials are often multiply published by lumping and splitting, than are those with negative results. This has produced an impression in the medical literature that the evidence for the effectiveness of antidepressants is much stronger than it actually is.
(e) Industry trials are more cited than are non-industry trials (Gorry 2015). This may be because when publication planners assign a manuscript to a ghostwriter, it appears that a list of references is frequently one of the key inputs, and companies have good marketing reasons to cite themselves (Sismondo 2020). However, the higher level of citation may be simply a result of the fact that pharmaceutical companies have much better resources for promoting their own trials than individual researchers have. For example, the companies employ thousands upon thousands of “key opinion leaders” to give talks to physicians, using prepared slide shows, on recent clinical research (Moynihan 2008; Sismondo 2018).

The pharmaceutical industry corrupts medical science and the medical literature through these mechanisms and many more (Sismondo 2018). In the ghost-management of research, much of the corruption does not happen via traditionally conceived conflicts of interest of independent medical researchers. Instead, it happens by more direct actions by drug companies and their agents, such as those listed in (a) to (e) above.

## Discussion: The Body of Medical Science

While it initially seems likely that medical science is corrupted by medical researchers’ conflicts of interest, that picture doesn’t capture at least some of what is going on. Instead, pharmaceutical companies create their own research and its own ways of disseminating that research, relying on structures and traditions of medical science to legitimate their work. While we could talk of companies as having conflicts of interest, it is more natural to talk of them as acting in their own interests.

In the pharmaceutical companies’ ghost-management of research, much of the corruption of medical science happens through a process of grafting. Grafts on plants make two bodies into one, typically allowing a fruiting part of a plant of value—to the horticulturist—to thrive by drawing on nutrients provided by a different plant’s rootstock. Grafting involves a carefully constructed parasitic relationship. Similarly, pharmaceutical companies add substantially to medical science, doing their own research, smoothly attaching it to medical science in a way that integrates it, and then nurturing it to make it predominate. Non-industry medical science provides legitimacy to the apparently similar additions. The effects of industry sponsorship of medical research are the results of prominent additions to the body of medical science, not the simple introduction of an element—such as funding—that infects what it touches.

Of course, the pharmaceutical industry is a huge one, and in some areas of medicine the grafts permeate or overwhelm everything else in the area. And it is likely that the grafts affect the bodies onto which they are grafted: industry science may, for example, create costly research norms that in turn create demand for more industry funding.

Like most systems that can be corrupted, medical science has never been pure or perfect. But the pharmaceutical industry can trade on the presumed innocence of medical research’s overriding goal: creating knowledge to benefit patient health. That is, some standard narratives of medical research attribute to it purity of heart, and a mere shortage of means that can be rectified by industry support.

In a very different context, Kierkegaard (1995: 76) writes: “As the world changes, the forms of corruption also gradually become more cunning, more difficult to point out.” In its corruption of medical science, the pharmaceutical industry has borne this out.

## Data Availability

Further inquiries can be directed to the corresponding author.
